# Fall armyworm (*Spodoptera frugiperda* Smith) feeding elicits differential defense responses in upland and lowland switchgrass

**DOI:** 10.1371/journal.pone.0218352

**Published:** 2019-06-13

**Authors:** Nathan A. Palmer, Saumik Basu, Tiffany Heng-Moss, Jeffrey D. Bradshaw, Gautam Sarath, Joe Louis

**Affiliations:** 1 Wheat, Sorghum, and Forage Research Unit, USDA-ARS, Lincoln, NE, United States of America; 2 Department of Entomology, University of Nebraska-Lincoln, Lincoln, NE, United States of America; 3 Department of Biochemistry, University of Nebraska-Lincoln, Lincoln, NE, United States of America; University of Kentucky, UNITED STATES

## Abstract

Switchgrass (*Panicum virgatum* L.) is a low input, high biomass perennial grass being developed for the bioenergy sector. Upland and lowland cultivars can differ in their responses to insect herbivory. Fall armyworm [FAW; *Spodoptera frugiperda* JE Smith (Lepidoptera: Noctuidae)] is a generalist pest of many plant species and can feed on switchgrass as well. Here, in two different trials, FAW larval mass were significantly reduced when fed on lowland cultivar Kanlow relative to larvae fed on upland cultivar Summer plants after 10 days. Hormone content of plants indicated elevated levels of the plant defense hormone jasmonic acid (JA) and its bioactive conjugate JA-Ile although significant differences were not observed. Conversely, the precursor to JA, 12-oxo-phytodienoic acid (OPDA) levels were significantly different between FAW fed Summer and Kanlow plants raising the possibility of differential signaling by OPDA in the two cultivars. Global transcriptome analysis revealed a stronger response in Kanlow plant relative to Summer plants. Among these changes were a preferential upregulation of several branches of terpenoid and phenylpropanoid biosynthesis in Kanlow plants suggesting that enhanced biosynthesis or accumulation of antifeedants could have negatively impacted FAW larval mass gain on Kanlow plants relative to Summer plants. A comparison of the switchgrass-FAW RNA-Seq dataset to those from maize-FAW and switchgrass-aphid interactions revealed that key components of plant responses to herbivory, including induction of JA biosynthesis, key transcription factors and JA-inducible genes were apparently conserved in switchgrass and maize. In addition, these data affirm earlier studies with FAW and aphids that the cultivar Kanlow can provide useful genetics for the breeding of switchgrass germplasm with improved insect resistance.

## Introduction

Switchgrass (*Panicum virgatum* L.) is an economically important C4 grass and considered as an emerging model for bioenergy crops [1]. However, switchgrass is not immune to attack and damage caused by insect pests. Switchgrass can act as a host for several feeding guilds of insect pests, including chewing, piercing-sucking, and cell-content feeding insects [2–7]. Pest pressure on switchgrass may pose a threat to breeding efforts attempting to develop insect-resistant switchgrass cultivars [8]. Thus, it is critical to understand how switchgrass exploits its endogenous defense mechanisms to enhance its immunity against insect assault.

Plants activate a suite of inducible defenses upon insect herbivory, which include both physical and chemical defenses [9–11]. Physical defenses include cuticle, trichomes, spines, and thorns, which potentially acts as a barrier to prevent insect feeding. Chemical defenses include several insecticidal compounds, such as saponin, cyanogenic glycosides, benzoxazinoids, cardenolides, chlorogenic acid, glucosinolates, and non-protein amino acids [12–17]. In addition, insect herbivory can induce several other insecticidal compounds such as phenolics, alkaloids, and proteases. For example, the maize genotype (Mp708) provides resistance to different feeding guilds of insects by rapidly accumulating Maize insect resistance1-Cysteine Protease (Mir1-CP), a papain-like protease [18–22]. These studies suggest that induced defenses in plants can have both direct or indirect consequences on the pest.

Previously, we identified resistant and susceptible cultivars in switchgrass against aphids [3, 23, 24]. In these studies, the tetraploid lowland ecotype (cv Kanlow) provided antibiosis (limits insect fecundity) mediated resistance to two different aphids: greenbugs (GB; *Schizaphis graminum* Rondani) and yellow sugarcane aphids (*Sipha flava*); whereas the upland ecotype (cv Summer) was tolerant to GB and susceptible to yellow sugarcane aphids [24]. Furthermore, an extensive study of switchgrass response to GB feeding provided a comprehensive view of how the switchgrass transcriptome changes in response to GB feeding and identified several transcription factors that could be driving these changes [25].

The fall armyworm [FAW; *Spodoptera frugiperda* JE Smith (Lepidoptera: Noctuidae)] is a generalist chewing insect that feeds on many grasses, including switchgrass [5]. In general, the lowland switchgrass cultivars may be more resistant to FAW compared to the upland cultivars [8]. In our study, the defense responses of two different switchgrass cultivars, Kanlow and Summer, to FAW herbivory was monitored using plant hormone analysis and RNA-Seq.

## Materials and methods

### Plant and insect materials

The two switchgrass cultivars used in this study were the lowland ecotype Kanlow and the upland ecotype Summer [26]. Plants were grown from seed in SC-10 Super Cell Single Cell Cone-tainers (3.8 cm x 21 cm plastic Cone-tainers; Stuewe & Sons, Inc., Corvallis, OR) containing a Fafard Growing Media (Mix No. 3B; Conrad Fafard, Awawam, MA). These plants were grown under 14L:10D, 400-W high-intensity lamps, 25 ± 7˚C at the University of Nebraska-Lincoln greenhouses. Plants were fertilized every two weeks with a soluble (20:10:20 N-P-K) fertilizer. Eight-week old plants were used for all the experiments. Newly hatched FAW larvae (‘corn strain’) were obtained from Benzon Research Inc., PA. Before infestation to plants, these larvae were kept in a growth chamber (25˚C; 14L:10D) for 4–6 h and allowed to acclimate.

### Insect bioassays

Two newly hatched FAW larvae were introduced per single switchgrass seedling, and each plant was individually caged with tubular plastic cages with vents covered with organdy fabric to confine the FAW on the infested plants. Twelve biological replicates were used for each cultivar. The larvae were allowed to feed for 10 days, at which time the cages were removed, and the larvae were recovered from individual plants and weighed. Uninfested control plants were similarly caged for 10 days. These bioassays were repeated two times with similar design.

### Tissue collection for phytohormone analysis and RNA-sequencing

Newly hatched FAW larvae were allowed to feed on eight-week old switchgrass plants. (two larvae per switchgrass plant) for 10 days. At 10 days post infestation (dpi), approximately 400 mg of shoot tissues surrounding the FAW feeding area were collected from the infested plants. Plants that were not infested with FAW were used as controls. The collected tissues were flash frozen in liquid N_2_ and stored at -80°C until analyzed. Samples were cryogenically ground and aliquots of ground tissues used for phytohormone analysis and RNA extraction, library preparation, and sequencing. Aliquots of 100 ± 2 mg of ground tissue were extracted for plant hormone analysis in methanol/acetonitrile (1:1 v/v) and analyzed by LC-MS/MS as described previously [27, 28]. Briefly, phytohormone analysis was carried out by the Proteomics & Metabolomics Facility at the Center for Biotechnology, University of Nebraska-Lincoln. The ground tissue was dissolved in cold methanol:acetonitrile (50:50, v/v) spiked with deuterium-labeled internal standards. After centrifugation at 16,000g, the supernatants were collected, and extraction of the pellet was repeated. The supernatants were pooled and dried down using a speed-vac. The pellets were redissolved in 200 μL of 15% methanol, and the supernatant analyzed for plant hormones using a combination of Shimadzu HPLC system interfaced with a Sciex QTRAP 6500+ mass spectrometer equipped with a TurboIonSpray (TIS) electrospray ion source. Analyst software (version 1.6.3) was used to control sample acquisition and data analysis. The QTRAP 6500+ mass spectrometer was tuned and calibrated according to the manufacturer’s recommendations. The hormones were detected using MRM transitions that were optimized using standards. The instrument was set up to acquire data in positive and negative ion switching modes. For quantification, an external standard curve was prepared using a series of standard samples containing different concentrations of unlabeled hormones and fixed concentrations of the deuterium-labeled standards mixture.

RNA was extracted from ~100 mg of ground tissue using a Direct-zol RNA kit (Zymo; Tustin, CA) following manufacture’s protocols. Total RNA was further processed at the University of Nebraska Medical Center Genomics Core Facility, Omaha, NE (www.unmc.edu/vcr/cores/vcr-cores/dna-sequencing). Briefly, 500 ng of total RNA was processed according to manufacturer supplied protocols for 3’-libarary generation (Lexogen QuantSeq 3’ mRNA-Seq library prep kit FWD for Illumina, Lexogen GmbH, Vienna, Austria), with a PCR amplification for 14 cycles. RNA and libraries were checked for quality using Qubit (ThermoFisher Scientific, Waltham, MA) and an Agilent 2100 Bioanalyzer (Agilent Technologies, Santa Clara, CA). Individual libraries were pooled with a loading concentration of 1.3pM and sequenced on NextSeq500 (Illumina, Inc., San Diego, CA), using a high output flowcell and sequencing kit to obtain 75 bp single read run. Run quality was monitored using Basespace (Illumina, Inc., San Diego, CA). The quality of the reads (QC30 average) was 92%, with an average output of 14 Million reads per 3’-library.

### Bioinformatic analyses

Demultiplexed raw reads were trimmed using bbduk, part of BBTools (https://jgi.doe.gov/data-and-tools/bbtools/), with the following parameters: k = 13, ktrim = r, useshortkmers = t, qtrim = r, trimq = 10, minlength = 20, mink = 5, ref = polyA.fa.gz,truseq_rna.fa.gz. Trimmed reads were then aligned to version 4.1 of the switchgrass genome (https://phytozome.jgi.doe.gov) using hisat2 [29]. Samtools was used to convert alignments to sorted BAM files [30], and gene expression counts were calculated for uniquely mapped reads using featureCounts [31].

NMDS plots were generated using the metaMDS function in the vegan-package [32] in R [33] with Euclidean distance measures. Differential expression analysis was done using the DESeq2 [34] package in R, with significance thresholds of false discovery rate < = 0.05 and log_2_ fold change > 1.0. The GeneOverlap package [35] in R was used to analyze KEGG pathway enrichment using a Fisher’s exact test approach.

### *Zea mays* orthology comparison

Switchgrass orthologs to *Zea mays* genes were identified by Inparanoid [36] analysis between the *Panicum virgatum* v4.1 and *Zea mays* Ensembl-18 reference genomes, included in Phytozome 12.1 (https://phytozome.jgi.doe.gov). Only 2:1, 2:2, 1:1, and 1:2 (switchgrass:maize) orthologs were included in the subsequent analyses. Maize genes up- or downregulated by FAW after 24 hours of infestation identified in Tzin et al. [37] were converted to their identified switchgrass orthologs and used to generate Venn diagrams.

### Statistical analysis

For insect bioassays and phytohormone analysis, analysis of variance (ANOVA) were performed using PROC GLM (SAS Institute). The normality and homogeneity of data were checked. Means were separated using Tukey’s honestly significant difference (HSD) tests (*P <* 0.05).

## Results

### FAW larval weights were significantly reduced when fed on Kanlow plants

**Fig 1:**
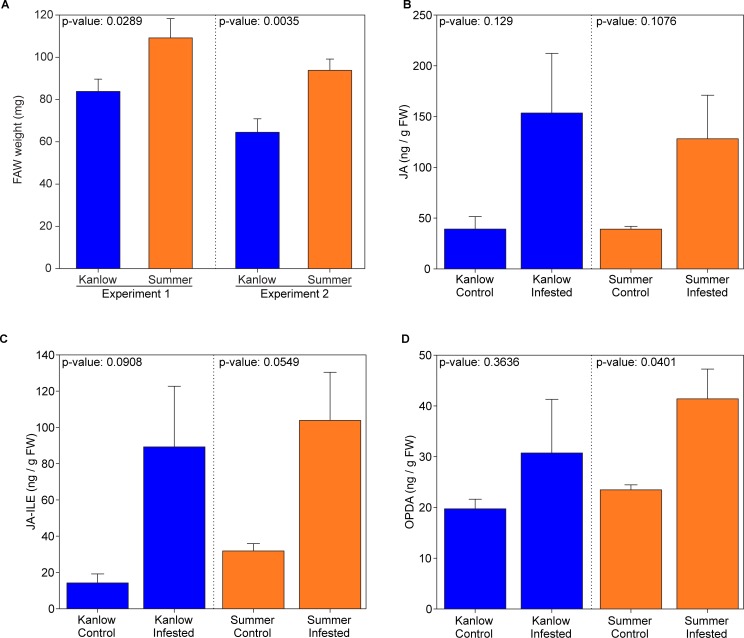
Bioassay and Phytohormones. (A) FAW weight 10 dpi on Kanlow (blue) or Summer (orange) seedlings. (B) Jasmonic acid (JA) quantification in Kanlow (blue) and Summer (orange) seedlings in uninfested control and FAW infested seedlings after 10 dpi. (C) JA-Ile quantification in Kanlow (blue) and Summer (orange) seedlings in uninfested control and FAW infested seedlings after 10 dpi. (D) OPDA quantification in Kanlow (blue) and Summer (orange) seedlings in uninfested control and FAW infested seedlings after 10 dpi. Different letters above the bars indicate values that are significantly different from each other (*P* < 0.05; Tukey’s test). Error bars represent SEM.

FAW larval mass was significantly reduced after 10 days of feeding on Kanlow seedlings relative to Summer seedlings in both experiments, although FAW larval mass was more reduced in Experiment 2 relative to Experiment 1 (Fig 1A). However, in both experiments feeding on Kanlow resulted in significantly reduced larval mass as compared to larvae feeding on Summer plants. This reduction in larval weights (Kanlow vs Summer) ranged from ~38 to 50% indicating a strong antibiosis interaction between FAW and Kanlow.

In both Kanlow and Summer plants, FAW herbivory increased tissue levels of JA, JA-Ile, and OPDA (Fig 1B, 1C and 1D), although significant differences (p-value < 0.05) between control and infested plants were only seen for OPDA in Summer plants.

### Kanlow plants had a stronger transcriptomic response to FAW feeding

**Fig 2:**
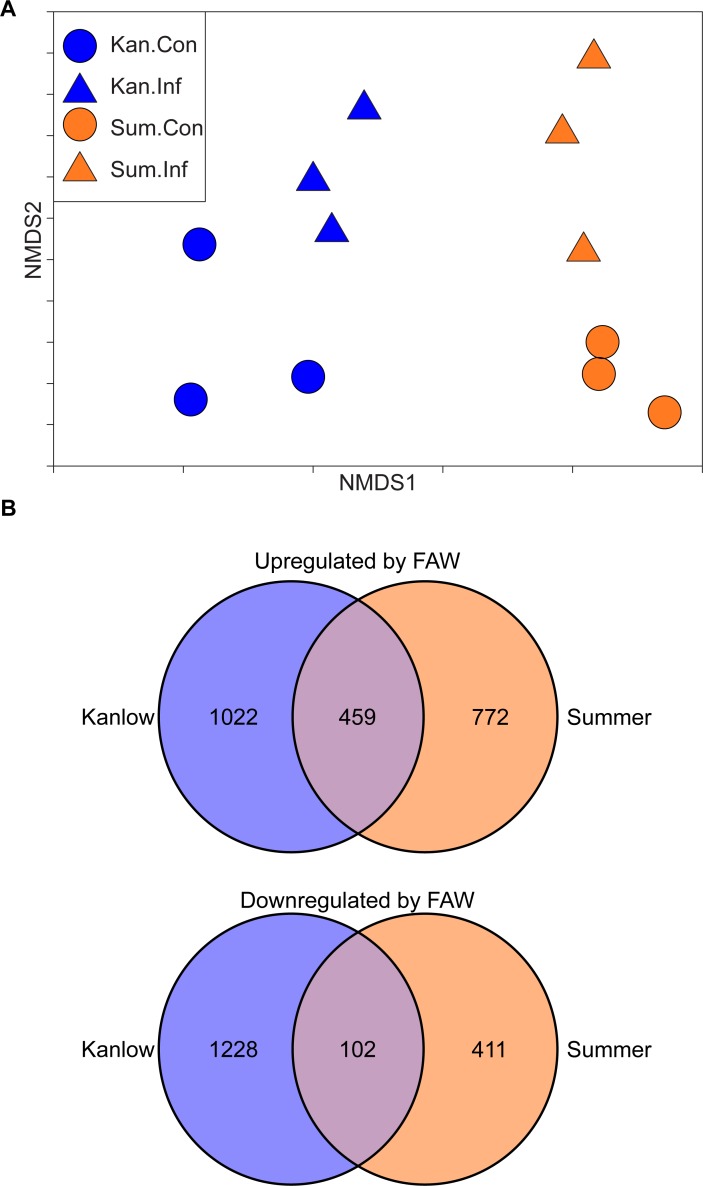
Transcriptome summary. (A) NMDS plot of all 12 RNA-Seq samples. (B) Venn diagrams showing the number of DEGs upregulated or downregulated by FAW in Kanlow and Summer switchgrass.

For both cultivars, FAW feeding changed the transcriptomes in a similar manner along NMDS axis 2 but maintained the ecotype differentiation along NMDS axis 1 (Fig 2A). Whole transcriptome analysis suggested that basal differences in gene expression existed between the two switchgrass ecotypes, and based on NMDS analysis, several similarities in gene expression could be anticipated in response to FAW feeding.

In total, 2253 and 1741 switchgrass genes were differentially up- and downregulated after ten days of FAW feeding (Fig 2B). A substantial portion of these differentially expressed genes (DEGs) were found in Kanlow plants, suggesting that Kanlow plants mounted a more robust transcriptomic response to FAW herbivory as compared to Summer plants. Both ecotypes shared approximately 20% of upregulated genes (459/2253) and approximately 6% of downregulated genes (102/1741). These data corroborated the NMDS analysis shown in Fig 2A. Differences in gene expression profiles of uninfested control plants of Kanlow and Summer were contributors to the differences in the numbers of DEGs that were unique to either ecotype under FAW herbivory, and the DEGs in common to FAW herbivory in either ecotype similarly pointed to a shared switchgrass defense response. These data provided evidence for both shared and ecotype-specific defense responses.

### DEG enrichment of pathways was more pronounced in FAW-infested Kanlow plants

**Fig 3:**
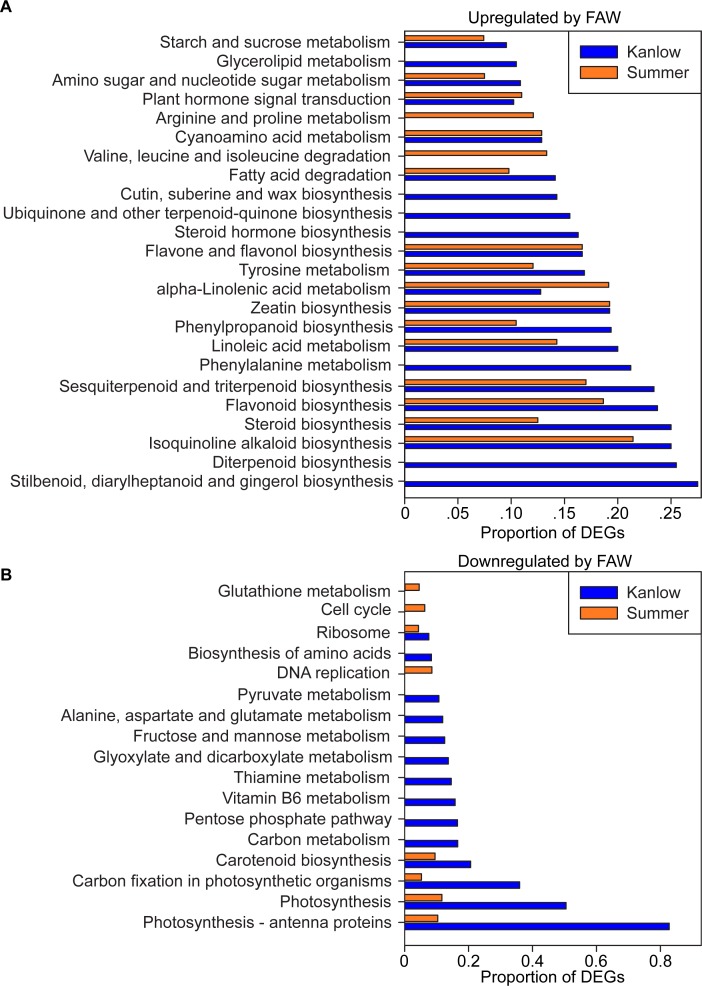
KEGG pathway enrichment in DEGs. (A) KEGG pathway significantly enriched (FDR < 0.05) in gene sets upregulated by FAW in Kanlow (blue) and Summer (orange) switchgrass. If the pathway was enriched, the proportion of DEGs in the gene set relative to the total number of expressed genes in the pathway is shown on the x-axis. (B) KEGG pathway significantly enriched (FDR < 0.05) in gene sets downregulated by FAW in Kanlow (blue) and Summer (orange) switchgrass.

Kyoto encyclopedia of genes and genomes (KEGG) [38] pathway enrichment analysis was performed to query putative metabolic associations of DEGs and to highlight similarities and differences in the ecotype responses to FAW herbivory. KEGG pathway enrichment indicated a greater association of DEGs in Kanlow plants with significantly enriched metabolic processes (Fig 3).

Among the 24 pathways that were upregulated by FAW infestation, 15 were common to both ecotypes, two were unique to infested Summer plants, and seven were unique to infested Kanlow plants (Fig 3A). Arginine and proline metabolism and valine, leucine, and isoleucine degradation were uniquely enriched in FAW-infested Summer plants. Among pathways enriched in both switchgrass ecotypes, α-linolenic acid metabolism associated DEGs were more abundant in Summer relative to Kanlow (18 vs 12), potentially linked to greater damage from FAW herbivory as indicated by higher larval mass for insects fed on Summer plants (see Fig 1A). Cyanoamino acid metabolism, plant hormone signal transduction, zeatin biosynthesis, and flavone and flavanol biosynthesis were equally represented by DEGs from both cultivars. All of the other KEGG metabolic pathways had higher representation of DEGs derived from Kanlow plants. Although DEGs associated with sesqiterpenoid and triterpenoid biosynthesis were found in both cultivars, those ascribed to phenylalanine metabolism, ubiquinone and other terpenoid-quinone biosynthesis, diterpenoid biosynthesis, and stilbenoid, diarylheptanoid, and gingerol biosynthesis were uniquely enriched in Kanlow plants (Fig 3A). Whether, anti-feedant and/or insecticidal compounds arising from these pathways contributed to the differential feeding of FAW is not currently known. Deamination of phenylalanine to 4-cinnamic acid (as part of phenylalanine metabolism) provides precursors for phenylpropanoid and terpenoid biosynthesis, and the fact that all of these pathways were significantly enriched in Kanlow plants suggests diversion of products of plant primary metabolism to defense compounds produced by switchgrass secondary metabolism.

A similar pattern of pathway enrichment occurred in the downregulated pathways (Fig 3B). Out of 17 KEGG pathways with significant downregulated DEG enrichment, five contained DEGs found in both switchgrasses in response to FAW feeding, three that were enriched only in Summer plants, and nine enriched only in Kanlow plants. Glutathione metabolism, cell cycle, and DNA replication were enriched in Summer, albeit, the proportion of DEGs was low (Fig 3B). Similarly, in the five pathways found to be enriched in common between the two switchgrasses, the proportion of DEGs associated with each pathway for Summer were generally much lower than those found in Kanlow. This differential enrichment of DEGs was especially evident in the four pathways responsible for primary carbon fixation, namely, carotenoid biosynthesis, carbon fixation in photosynthetic organisms, photosynthesis and photosynthesis–antenna proteins. Plausibly, the strong downregulation of photosynthetic and pigment biosynthesis-related pathways in Kanlow plants, combined with a significant upregulation of terpenoid biosynthesis, could be contributors to overall better defense responses of Kanlow relative to Summer against FAW herbivory.

### Core genes present in switchgrass responses to insect herbivory

Genes up/downregulated by FAW in switchgrass in this current study were compared to similarly annotated genes in previously published transcriptomic datasets on switchgrass (Summer) responses to GB herbivory [25] and switchgrass orthologs of maize (*Zea mays*. L. ssp. mays) responses to FAW herbivory [37]. Of significant interest were those DEGs found in common between the four different datasets, with the expectation that these genes were part of a core set of genes important to switchgrass defense response to differ guilds of insects, and possibly part of similar networks in other grasses, such as maize.

**Fig 4:**
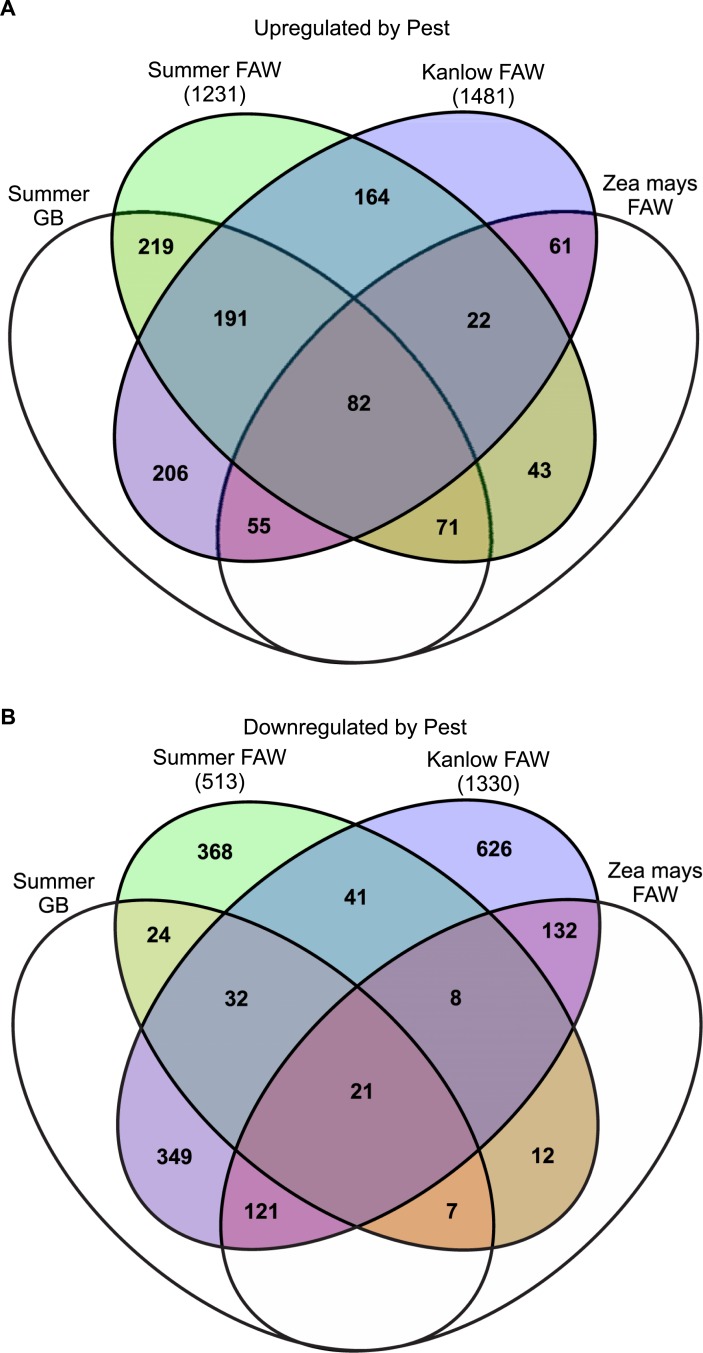
Meta-analysis of genes induced by insect pests. Venn diagrams showing the overlap of DEGs in this study compared to DEGs identified in Donze-Reiner et al. [25] resulting from GB feeding on Summer switchgrass and switchgrass orthologs to DEGs identified in Tzin et al. [37] resulting from FAW feeding on *Zea mays* after 24 hours.

Analysis of the 1231 and 1481 upregulated DEGs from Summer and Kanlow with the same or similar genes from switchgrass and maize documented a complex pattern of overlaps (Fig 4A). A majority of Kanlow genes (~47%) upregulated by FAW herbivory were unique to Kanlow. In contrast, approximately 36% of upregulated genes were unique to the Summer x FAW dataset. About similar numbers of upregulated DEGs 219 and 206, were shared in common between the Summer x FAW and Summer x GB, and Kanlow x FAW and Summer x GB datasets respectively. Although fewer total DEGs were found between the switchgrass datasets and Maize x FAW dataset, 82 DEGs were shared in common between all the comparisons (Fig 4A). Functional annotations of these DEGs are provided in S1 Data.

Patterns of common and unique DEGs that were downregulated in response to a pest are shown in Fig 4B. As observed for upregulated DEGs, a majority of the downregulated genes in response to FAW herbivory (626/1330) were unique to Kanlow and to Summer (368/513). However, unlike the patterns observed with the upregulated DEGs, much greater numbers of genes downregulated by FAW were shared with the Summer x GB dataset, especially in Kanlow (349/1330), and between Kanlow x FAW, Summer x GB, and maize x FAW (121). A smaller number of downregulated DEGs (21) was shared between all of comparisons (Fig 4B).

Analysis of the DEGs shared in common to the four datasets indicated the potential for conserved defense response to GB and FAW. Of the 82 upregulated DEGs 14 were transcription factors, consisting of five WRKYs, three ERFs, two zinc-finger proteins, two heat shock factor 4, one MYB, and one scarecrow-like factor. Other upregulated genes included those involved in JA biosynthesis, such as those encoding lipoxgenase 2, oxophytodienoate-reductase 3, OPC-8:0 CoA ligase 1, allene oxide synthase, and allene oxide cyclase 3; redox-related genes encoding peroxidases and cytochrome b5; and several defense-associated genes encoding, chitinase, β-glucanase, and PR proteins, among others (S1 Data).

Downregulated DEGs shared in common were mostly associated with stress, plastids, and carbon fixation. Only one transcription factor gene was downregulated in common and it encoded a nuclear factor Y subunit 4 ortholog. Stress-related genes included zeaxanthin epoxidase, UDP-glucosyltransferase, two chaperones, and ascorbate peroxidase. Several genes encoding integral plastid proteins were also significantly downregulated by insect pressure in switchgrass and maize and included an ortholog to chlororespiratory reduction 6 which is required for efficient assembly and stabilization of the photosystem I NAD(P)H dehydrogenase complex [39]. Similarly, pyruvate orthophosphate dikinase (PPDK), a critical gene involved in carbon assimilation in C_4_ plants, was downregulated, along with a phosphoglucomutase and a plastid-localized glyceraldehyde-3-phosphate dehydrogenase.

## Discussion

Switchgrass consists of upland and lowland ecotypes of variable ploidy that differ in their responses to a variety of biotic stressors [26, 40–45]. Lowland switchgrass tetraploids have greater biomass yields relative to upland tetraploids, but lack robust winter survival, precluding their deployment as a bioenergy crop in more northern latitudes of the USA [46]. However, hybrids between the lowland cultivar Kanlow and the upland cultivar Summer are heterotic for biomass yields and have better winter survival than Kanlow plants [47, 48]. As a consequence Kanlow and Summer have become mainstays of the switchgrass breeding program at the ARS-Lincoln, NE location [49, 50]. The responses of switchgrass ecotypes and cultivars (lowland and upland) to different aphids have been documented [3, 23, 24, 26], but the mechanistic details of these differential responses have not been elucidated in switchgrass-FAW system. It is well known that JA or JA-dependent defenses contribute to plant defense against chewing herbivores [9]. Significant differences were observed in the levels of the JA intermediate, OPDA[51] in FAW-damaged Summer plants, but not in Kanlow plants, suggesting that changes in the relative cellular levels of JA and OPDA could be one facet driving differential responses of these two cultivars to insect pests. High OPDA levels favored the growth of cabbage loopers (*Trichoplusia ni*) when reared on the Arabidopsis *opr3* plants. Arabidopsis *opr3* plants accumulate elevated levels of OPDA, indicating that the OPDA could act as a susceptibility factor against chewing herbivores [52].

Recently, Donze-Reiner et al. [25] investigated the temporal defense responses of Summer plants in response to phloem sap-feeding aphid, GB. Interestingly, diverse sets of defense related genes were found to be activated at different time points. For example, GB feeding resulted in an early activation of various ROS pathway genes and increased the production of defense-related proteins over time, followed by a later recovery phase leading to dampening of the defense responses [25]. In this current study, transcript abundance data indicated that FAW-herbivory likely upregulated biosynthetic pathways that lead to the production of several secondary metabolites in Kanlow plants. Plausibly, accumulation of terpenoids and other secondary defense metabolites could have negatively impacted FAW growth and development on Kanlow plants relative to Summer plants. Although, increased production of terpenoid compounds have been found to have insecticidal activities across many crops [53, 54], the exact mechanism of differential antifeedant/insecticidal activity in these two switchgrass varieties are yet to be determined.

Plants can modulate their rate of photosynthesis, source-sink relationships, nutrient allocation, carbohydrate metabolism, and nutrient transport upon insect attack [4, 55–58]. Similarly, transcriptional evidence suggested that Kanlow plants altered nutrient/carbon allocations potentially depriving insects of nutrients needed for growth and development. These data combined with the potential of increased defense compound biosynthesis could underpin the superior resistance responses of Kanlow plants relative to Summer plants to FAW herbivory.

A comparison of genes commonly up/down regulated by FAW (this study) to maize [37] and in switchgrass infested with GB [25] indicated several commonalities between these systems. As noted earlier, many of the genes downregulated across these interactions were related to photosynthesis. Both reductions in C-assimilation as well as changes in partitioning of carbohydrates between sugars and starch appear to be well conserved mechanisms in plant-herbivorous insect interactions [4, 58–60], likely mitigating the loss of nutrients, and improving plant performance. Genes functionally annotated as zeaxanthin epoxidase (ZEP) homologs to Arabidopsis ABA1 (AT5G67030) were downregulated in these interactions. ZEP is plastid-localized, required for ABA and xanthophyll biosynthesis, and appears to be part of plant stress responses [61]. Downregulation of ZEP in switchgrass and maize plants might be reflective of the predicted changes occurring in plastids.

Several transcription factors were upregulated in common, again suggesting similarities in the basal defense responses in switchgrass and maize to insect herbivores. Orthologs of the Arabidopsis zinc-finger 1 (ZF1; AT5G67450) were induced by FAW and aphids. ZF1 is a negative regulator of ABA-repressed genes and functions as a transcriptional repressor when plants are exposed to a variety of stress [62, 63]. Hormonal levels including ABA can change in response to herbivory [64]. Transcriptional evidence for downregulation of chloroplastic functions suggest that changes in plastid metabolism might also inhibit ABA biosynthesis in leaves, potentially triggering diverse signaling circuits, such as those related to ZF1, among others.

WRKYs are another important class of defense-related transcription factors that were induced by herbivory in the two grasses. These grass WRKYs were orthologous to Arabidopsis WRKY28, 51, 55, 72, and 75 respectively. Arabidopsis WRKY28, 51, 72, and 75 have been implicated in plant defense [65–67]. Interestingly, WRKY51 is upstream of initiation of JA biosynthesis in Arabidopsis and activated by the intracellular increase of Ca^2+^ that occurs from insect herbivory [67]. Changes in Ca^2+^ levels upon aphid feeding have been shown to be important in Arabidopsis [68] and linked to initial responses of switchgrass to GB herbivory [25]. These data suggest that Ca^2+^-linked signaling components are likely conserved in switchgrass, maize, and Arabidopsis.

The link between WRKY51 and JA biosynthesis also appears to be conserved as well, since transcripts for several genes required for JA biosynthesis were upregulated upon FAW feeding on maize and switchgrass, as well as by GB herbivory of Summer switchgrass. JA levels were elevated in switchgrass plant infested with FAW, and several downstream genes induced by JA were upregulated in all four dataset comparisons. These JA-regulated genes included uclacyanins [69], JAZ1 [70], and several defense-related genes including chitinases and peroxidases that respond positively to JA [71].

In conclusion, our results support the recent observations of low FAW growth rates when fed on Kanlow plants [8] and extend these findings at the transcriptional level. The observed differential defense responses of two different switchgrass cultivars to FAW herbivory indicate that the lowland cultivar Kanlow mounted a more robust response with potential activation of pathways that could lead to the production of antifeedants, as compared to the upland Summer cultivar. These data affirm earlier studies with aphids that the cultivar Kanlow can provide useful genetics for the breeding of switchgrass germplasm with improved insect resistance.

## Supporting information

S1 Data Gene annotations. (XLSX)
